# In Situ Activating Strategy to Significantly Boost Oxygen Electrocatalysis of Commercial Carbon Cloth for Flexible and Rechargeable Zn‐Air Batteries

**DOI:** 10.1002/advs.201800760

**Published:** 2018-10-18

**Authors:** Zhe Zhao, Zhongke Yuan, Zhengsong Fang, Junhua Jian, Jing Li, Meijia Yang, Chunshao Mo, You Zhang, Xuanhe Hu, Ping Li, Shuangyin Wang, Wei Hong, Zhikun Zheng, Gangfeng Ouyang, Xudong Chen, Dingshan Yu

**Affiliations:** ^1^ Key Laboratory for Polymeric Composite and Functional Materials of Ministry of Education Key Laboratory of High Performance Polymer‐Based Composites of Guangdong Province School of Chemistry Sun Yat‐Sen University Guangzhou 510275 China; ^2^ College of Chemistry and Chemical Engineering Hunan University Changsha 410082 China; ^3^ School of Chemistry Sun Yat‐Sen University Guangzhou 510275 China

**Keywords:** bifunctional oxygen electrodes, flexible Zn‐air batteries, heteroatom doping, porous carbon, rechargeable Zn‐air batteries

## Abstract

An in situ strategy to simultaneously boost oxygen reduction and oxygen evolution (ORR/OER) activities of commercial carbon textiles is reported and the direct use of such ubiquitous raw material as low‐cost, efficient, robust, self‐supporting, and bifunctional air electrodes in rechargeable Zn‐air batteries is demonstrated. This strategy not only furnishes carbon textiles with a large surface area and hierarchical meso‐microporosity, but also enables efficient dual‐doping of N and S into carbon skeletons while retaining high conductivity and stable monolithic structures. Thus, although original carbon textile has rather poor catalytic activity, the activated textiles without loading other active materials yield effective ORR/OER bifunctionality and stability with a much lower reversible overpotential (0.87 V) than those of Pt/C (1.10 V) and RuO_2_ (1.02 V) and many reported metal‐free bifunctional catalysts. Importantly, they can concurrently function as current collectors and as ORR/OER catalysts for rechargeable aqueous and flexible solid‐state Zn‐air batteries, showing excellent cell performance, long lifetime, and high flexibility.

Ever‐increasing energy demands call for the development of alternative energy systems with satisfactory energy density.^1^, ^2^, ^3^ Among diverse candidates, rechargeable Zn‐air batteries are of particular interest due to ultrahigh theoretical energy density (1086 Wh kg^−1^), high safety, and low cost, yet their energy efficiency is limited by sluggish oxygen reduction/evolution reactions (ORR/OER) at air electrodes.^1^, ^2^, ^3^, ^4^, ^5^ So far, the best catalysts for ORR and OER are Pt and Ir/Ru based materials, respectively. But these noble metal catalysts alone are active for ORR or OER rather than both, together with high cost and poor stability, hindering their widespread deployment.^1^ Thus, exploring efficient, durable yet inexpensive bifunctional ORR/OER catalysts is of paramount importance for advanced rechargeable Zn‐air batteries.^4^, ^5^, ^6^, ^7^, ^8^, ^9^, ^10^, ^11^, ^12^, ^13^

Currently, metal‐free carbonaceous materials have received intense attention for oxygen catalysis due to low cost, high surface area, excellent electronic conductivity, and good stability.^10^, ^14^, ^15^, ^16^, ^17^, ^18^, ^19^, ^20^, ^21^, ^22^ By incorporating heteroatoms or edge defects or functional groups, some carbon materials were capable of simultaneously catalyzing both ORR and OER.^14^, ^23^, ^24^ Furthermore, their metal‐free nature can avert metal ion release, leading to reduced environmental effect. Despite great advance, most literature‐reported carbonaceous catalysts were produced in the powdery form and the electrode assemblies often involved elaborate film‐coating process with the aid of additives such as conductive support and insulating polymer binders.^4^, ^5^, ^6^, ^7^, ^8^, ^9^, ^25^, ^26^, ^27^, ^28^, ^29^ This inevitably leads to enlarged interfacial resistance, the blocking of catalytic sites, and possible mechanical peeling of catalysts from electrodes, which thereby compromises the catalytic efficiency and durability. Particularly, most literature‐reported two‐electrode Zn‐air batteries using metal‐free catalysts show unsatisfactory cycling life.^4^ The cycle stability could be enhanced by the tri‐electrode configuration. However, the complicated structure and operation inevitably restrict its practical application to a certain extent.^9^ Thus, to surmount the above limitation, it is highly desirable to develop robust, self‐standing, additive‐free, and metal‐free ORR and OER bifunctional electrocatalysts.^30^

Carbon cloth (CC) is a commonly seen and cheap textile with high conductivity and mechanical flexibility and is often used as the catalyst support/current collectors in various energy devices.^31^ However, owing to ultralow surface area and the lack of active sites, pristine CC has rather poor catalytic activity.^31^ Encouragingly, a few studies showed that several strategies such as acid treatment,^31^, ^32^ electrochemical activation,^33^ or Ar plasma,^18^ could activate the carbon surface and improve the electrochemical performance of original carbon fibers or CC while keeping monolithic structures and high conductivity, which makes it possible to enable the direct utilization of CC as self‐supporting and additive‐free catalytic electrodes by rational surface engineering. However, recent reports focus on the utilization of carbon fibers or CC only for one single reaction (either ORR or OER) and the available strategies are often effective for one oxygen reaction rather than both.^32^, ^34^ To date, only two reports were found to use carbon fibers as a bifunctional catalyst for both ORR and OER enabled by incorporating defects or N/O dopants into the carbon framework, yet the demonstrated OER activities were very low with high overpotentials at 10 mA cm^−2^ (450 and 558 mV),^18^, ^35^ which eventually led to poor ORR/OER bifunctionality and impracticality of real applications. The main reason possibly lies in low density of accessible and reversible oxygen catalytic sites and undesired porous structures. To our knowledge, no strategies are available to simultaneously improve ORR and OER performance of CC up to a high level. In fact, owing to different reaction mechanisms and kinetics for two oxygen reactions, integrating efficient ORR/OER bifunctionality and durability into one single material is not simple task. Thus, further boosting the bifunctional ORR/OER activities of CC and expediting the direct use of such common material as robust, self‐supporting, and bifunctional air electrodes in practical rechargeable Zn‐air batteries is highly desired yet a big challenge.

In response, we report an effective strategy to simultaneously boost ORR and OER activities of commercial CC by combined in situ texturizing and surface engineering and demonstrate the direct utilization of CC as low‐cost, efficient, robust, self‐supporting, and bifunctional air electrodes in practical rechargeable Zn‐air batteries. Our approach not only endows CC with a large surface area and hierarchical meso‐microporosity for improved mass transport and increased active surface area, but also enables dual doping of S and N into carbon skeletons to afford high density of exposed ORR/OER active sites while inducing a synergistic activity enhancement, which overcomes the limitation of previous strategies which are useful for merely one oxygen reaction rather than both. Different from previously reported N, S codoped carbon catalysts produced in the powdery form, our activated CC can serve as a new kind of “all‐in‐one” air electrodes that integrates the function of current collectors, good flexibility, and excellent ORR/OER bifunctional catalytic properties into one material. It is noted that our newly developed flexible CC catalyst combining hierarchical porosity and effective N, S codoping has never been reported before. Although native CC is electrochemically inactive, our activated CC without loading other active materials yields excellent ORR/OER bifunctionality and durability with a much lower reversible overpotential surpassing Pt/C and RuO_2_ and many existing metal‐free bifunctional catalysts. Importantly, when using activated CC as air electrodes without binders and extra current collectors, rechargeable aqueous and flexible solid‐state Zn‐air batteries show excellent cell performance, long lifetime, and high mechanical stability. Particularly, aqueous two‐electrode cell achieves superb cycling stability over 1000 cycles, superior to most literature‐reported two‐electrode Zn‐air systems using those powdery catalysts (typically <200 cycles) under similar conditions (Table S2, Supporting Information).

Our approach to boost the electrochemical performance of CC involves multiple steps: carbothermic reaction, hydrothermal processing initiated sulfur doping, and NH_3_ plasma treatment. In previous studies, carbon is widely used as a reducing agent to extract elemental metals (i.e., Zn) from metal oxides (i.e., ZnO) by carbothermic reduction.^21^, ^36^ Encouraged by this fact, one can conceive otherwise. If metal oxide rather than carbon is utilized as a sacrificial agent and uniformly coated onto CC, it is possible to activate/or roughen the carbon surface and thus improve the surface area and porosity of CC by the reduction of metal oxides and subsequent metal evaporation by carbothermic reaction at a desirable temperature. Considering that Zn has a relative low boiling point (908 °C),^21^ we first loaded clean CC into the autoclave, followed by the ZnO deposition on CC via hydrothermal treatment. The presence of the ZnO coating layer was verified by scanning electron microscopic (SEM) observation with element mapping and X‐ray diffraction (XRD) analysis (Figure S1, Supporting Information). Subsequent thermal treatment of the ZnO coated CC in Ar at 950 °C was done to initiate carbothermic reaction and eventually afford in situ textured porous CC (T‐CC), wherein carbon fiber surfaces were roughened with a pore network formed on the fiber wall (Figure S2, Supporting Information), caused by the evaporation of Zn‐ and O‐related species during annealing. Afterward, sulfur was incorporated to produce S‐doped porous CC (S‐CC) by hydrothermal treatment at 180 °C with thiourea (THU), followed by pyrolysis in Ar with benzyl disulfide (BDS). Finally, NH_3_ plasma treatment was performed to further dope N into S‐CC, yielding N, S codoped porous CC (N, S‐CC). It is noted that NH_3_ plasma could not only realize effective N doping and remove residual oxygen‐containing groups for improved electron transfer, but also etch the CC surface to engender more defect sites (Figure S3, Supporting Information), as also demonstrated in previous work.^37^, ^38^ As a control, N‐doped porous CC (N‐CC) and S‐doped porous CC (S‐CC) were also prepared following similar procedures (Figure S4, see details in the Supporting Information). Similar to T‐CC, SEM imaging of N, S‐CC reveals a highly porous structure consisting of numerous cracks, grooves, and craters on the carbon surface (**Figure** **1**c,d), in stark contrast to original CC with smooth surfaces (Figure 1b). The elemental mapping analysis of N, S‐CC illustrates uniform distribution of C, N, S elements on individual carbon fibers (Figure 1e). Transmission electron microscopy (TEM) observation uncovers exfoliated carbon segments attached to the intact fiber core, producing unique shell–core structures wherein the shell was loose and porous with a thickness of 100–150 nm (Figure 1f,g). Notably, in our approach, the macroscopic morphology, mechanical flexibility, and high conductivity of original CC are still maintained after activation treatment (Figures S5 and S6, Supporting Information). Particularly, the resistance of N, S‐CC remains almost unchanged under cyclic bending tests, implying excellent electromechanical stability (Figure S7, Supporting Information). After activation, N, S‐CC shows an excellent wettability, which facilitates the efficient access of electrolytes (Movie S1, Supporting Information). Furthermore, X‐ray diffraction analysis (Figure S8, Supporting Information) indicates that the crystalline structure of activated CC gives minor changes relative to pristine CC.

**Figure 1:**
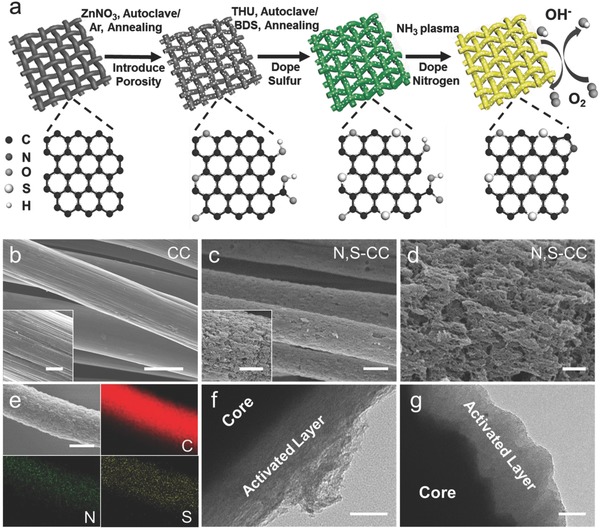
a) Schematic illustration for the preparation of N, S‐CC by three‐step in situ activation process. b,c) Typical SEM images of bare CC and N, S‐CC. d) Enlarged SEM image of the N, S‐CC surface. e) SEM image of individual N, S‐CC fiber and the corresponding elemental mapping images of C, N, and S. f,g) TEM images of individual N, S‐CC fiber collected at the edge and cross section. Scale bars: (b) 10 µm (inset: 2 µm); (c) 5 µm (inset: 2 µm); (d) 500 nm; (e) 5 µm; (f) 50 nm; (g) 100 nm.

Nitrogen adsorption–desorption isotherms and the corresponding pore size distribution curves shown in **Figure** **2**a,b unfold hierarchically micro‐mesoporous structured feature of N, S‐CC. Remarkably, N, S‐CC yields a specific surface area (SSA) of 146.2 m^2^ g^−1^ and a pore volume of 0.24 m^3^ g^−1^, both of which are significantly larger than those of CC (14.5 and 0.04 m^3^ g^−1^) and superior to previously reported CC with only mesoporous structures produced by acid treatment or electrochemical activation.^18^, ^31^, ^32^, ^33^ Unique hierarchical meso‐microporosity with a large SSA for N, S‐CC not only favors the exposure of more active sites and but also facilitates improved mass transfer during oxygen reactions. X‐ray photoelectron spectroscopy (XPS) in Figure 2c indicates the presence of C, N, S, and O elements in N, S‐CC. The high‐resolution N1s spectrum for N, S‐CC can be deconvoluted into three components corresponding to pyridinic‐N (398.9 eV), pyrrolic‐N (399.9 eV), and graphitic‐N (400.8 eV),^21^ while the S2p spectrum features two doublet peaks at 163.8 and 165.1 eV, respectively, attributable to the 2p_3/2_ and 2p_1/2_ transitions for C—S—C.^20^ The C1s spectrum can be split into multiple subpeaks, corresponding to C—S (283.4 eV) and C—N (285.2 eV), further verifying the successful incorporation of N and S into carbon skeletons. Meanwhile, a weak C—O subpeak (286.4 eV) was also observed in N, S‐CC similar to pristine CC (Figure S9, Supporting Information). This indicates that oxygen‐carrying species in N, S‐CC were largely removed after activation treatment, which is also evidenced by the high‐resolution O1s spectrum (Figure S10, Supporting Information). Raman spectra of original CC and N, S‐CC reveal the typical D and G peaks at 1350 and 1590 cm^−1^, respectively (Figure S3, Supporting Information).^37^, ^38^ Generally, the intensity ratio of D to G band (*I*_D_/*I*_G_) is indicative of the amount of defect sites and disordered structures.^20^ The N, S‐CC gives a higher *I*_D_/*I*_G_ value (1.13) relative to CC (0.8), indicating more defect sites existed in N, S‐CC caused by the increased porosity and the incorporation of extra N and S atoms.

**Figure 2:**
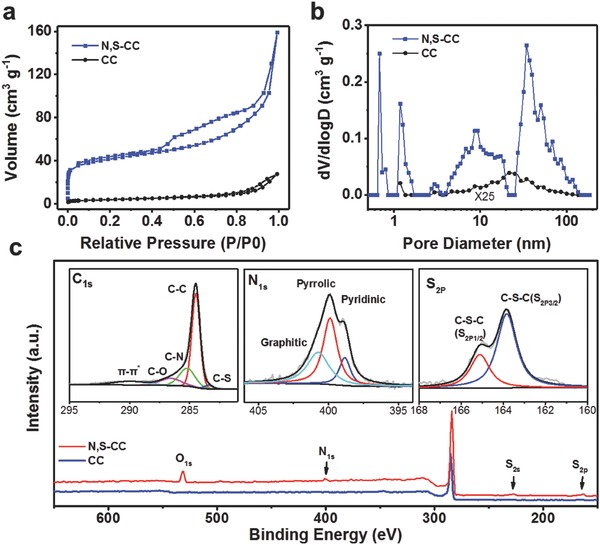
a) Nitrogen adsorption–desorption isotherms and b) the corresponding pore size distribution curves of CC (*y*‐axis scale: ×25) and N, S‐CC. c) XPS survey of CC and N, S‐CC. The inset shows high‐resolution XPS spectra of C_1s_, N_1s_, and S_2p_ core levels in N, S‐CC.

The OER performance of N, S‐CC was first assessed in 1 m KOH electrolyte and compared with the control samples. Similar to previous reports,^4^, ^7^, ^22^ the applied potential range is 1.2–1.75 V versus reversible hydrogen electrode (RHE), avoiding possible electrooxidation of carbon at overhigh potential. **Figure** **3**a presents the polarization curves of different catalysts with 100% *iR* compensation. As observed, N, S‐CC shows the best OER activity with a low onset potential of ≈1.4 V versus RHE and a high catalytic current density (*j*_GSA_, GSA = geometric surface area) over a wide potential range. Particularly, the overpotential (η) of N, S‐CC acquired at 10 mA cm^−2^ (Figure 3b) is as low as 360 mV, much superior to those obtained for other samples including original CC (470 mV), N‐CC (429 mV), S‐CC (422 mV), and most of the existing metal‐free catalysts (Table S1, Supporting Information) and even comparable to that of the benchmark RuO_2_ loaded on CC (320 mV, Figure S11, Supporting Information). Furthermore, N, S‐CC delivers a large current density of ≈22 mA cm^−2^ at η = 420 mV, which is ≈4 times higher than that of CC (≈5 mA cm^−2^), manifesting a large activity improvement upon activation. N, S‐CC also gives a smaller Tafel slope with respect to the counterpart samples (Figure S12, Supporting Information), implying more favorable OER kinetics. Electrochemical impedance results further reveal that the N, S‐CC catalyst has the smallest charge transfer resistance among all investigated systems (Figure S13, Supporting Information), signifying rapid electron transfer rate for N, S‐CC. It is noted that monodoped porous N‐CC or S‐CC and dual‐doped porous N, S‐CC possess similar contents of N and S (Figures S14 and S15, Supporting Information) and similar porous structures (Figure S4, Supporting Information), thereby highlighting a synergistic role of N and S dopants in enhancing the OER activity of N, S‐CC. The OER reaction mechanism was probed by a rotating ring‐disk electrode (RRDE) in 1 m KOH. Figure S16 (Supporting Information) exhibits a rather low current density for H_2_O_2_ oxidation at the Pt ring electrode, indicating a nearly four‐electron water oxidation pathway occurred at N, S‐CC. Moreover, the Faradaic efficiency of the N, S‐CC electrode was determined to be ≈97% by the volumetric method at 10 mA cm^−2^, verifying that the anodic current mainly comes from water oxidation rather than carbon oxidation. This is further evidenced by gas chromatography analysis that no possible oxidation products (i.e., CO, CO_2_) were detected. The durability of N, S‐CC was examined through chronopotentiometric measurements in 1 m KOH. As seen in Figure S17 (Supporting Information), the potential of the N, S‐CC electrode to afford 10 mA cm^−2^ remains rather stable over 25 000 s.

**Figure 3:**
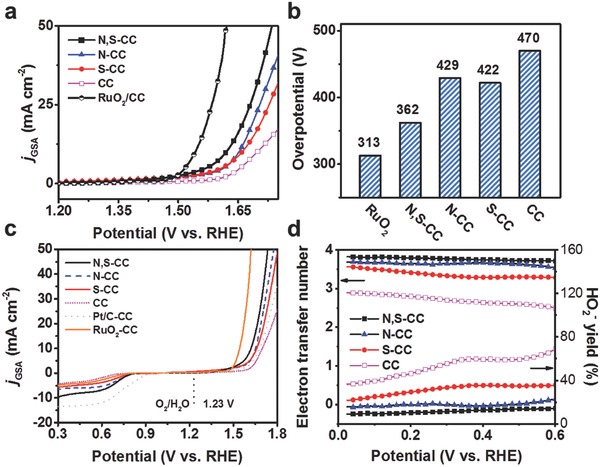
a) OER polarization curves and b) the overpotentials at 10 mA cm^−2^ of CC, S‐CC, N‐CC, N, S‐CC, and RuO_2_‐CC in 1 m KOH. c) Overall polarization curves of different catalysts within the ORR and OER potential window in 1 m KOH. d) HO_2_^−^ yield ratio and electron transfer number (*n*) during ORR of different catalysts in 1 m KOH.

Interestingly, N, S‐CC also yields the highest ORR activity among all investigated samples in 1 m KOH, which is evidenced by a more positive onset potential (≈0.83 V) and a substantially higher limiting current density (≈9.5 mA cm^−2^) relative to other samples including bare CC (0.69 V, 4.4 mA cm^−2^), N‐CC (0.81 V, 6.5 mA cm^−2^), and S‐CC (0.78 V, 5.7 mA cm^−2^) derived from polarization curves shown in Figure 3c and Figure S18 (Supporting Information). RRDE test unravels the HO_2_^−^ yield of 8.9–14% with electron transfer number (*n*) ranging from 3.72 to 3.82 for the best N, S‐CC catalyst, close to that of the benchmark Pt/C loaded CC (Pt/C‐CC, HO_2_^−^, 6.6–12%; *n* = 3.75–3.87, Figure S19, Supporting Information). This result signifies a desirable four‐electron transfer pathway over N, S‐CC. Moreover, N, S‐CC also presents much better durability in continuous reduction reaction in stark contrast to Pt/C‐CC (Figure S20, Supporting Information). Overall oxygen electrode performance was assessed by the potential gap (Δ*E*) between the OER potential acquired at 10 mA cm^−2^ and the half‐wave potential in ORR. In general, the smaller Δ*E* value is indicative of higher bifunctional activity.^4^ As shown in Figure 3c, N, S‐CC without loading other materials yields a low Δ*E* value of 0.87 V in 1 m KOH, outperforming the benchmark catalyst loaded CC such as Pt/C‐CC and RuO_2_‐CC (1.10 and 1.02 V) and many reported metal‐free bifunctional oxygen catalysts.^6^, ^18^, ^39^

In light of excellent bifunctional ORR/OER activity and high conductivity of freestanding N, S‐CC, we assembled an aqueous two‐electrode rechargeable Zn‐air battery utilizing a zinc foil as the anode and N, S‐CC directly as the additive‐free air electrode (**Figure** **4**a), avoiding complicated electrode preparation processes in previous Zn‐air batteries using those powdery catalysts.^4^, ^5^, ^6^, ^7^, ^8^, ^9^, ^10^, ^25^, ^26^, ^27^, ^28^ The Zn‐air battery based on N, S‐CC presents a high open‐circuit potential of 1.36 V (Figure S21, Supporting Information), which approaches the theoretical value of Zn‐air batteries. Furthermore, our battery delivers similar charge–discharge voltage gap with precious metal catalyst couple (Pt/C+RuO_2_) and a peak power density of 42 mW cm^−2^ (Figure 4b). As shown in Figure S22a (Supporting Information), the galvanostatic discharge voltage plateaus of battery based on N, S‐CC are comparable to that of Pt/C+RuO_2_, and vary slightly with increasing current densities from 2 to 10 mA cm^−2^, which indicated the excellent rate performance of our battery. When normalized to the mass of consumed zinc electrode (Figure 4c), the N, S‐CC‐based battery yields a high specific capacity of 715 mAh g_Zn_^−1^ that is close to the Pt/C+RuO_2_ counterpart of 746 mAh g_Zn_^−1^ at 5 mA cm^−2^ (≈87% utilization of the theoretical capacity of 820 mAh g_Zn_^−1^), corresponding to a high energy density of ≈829 Wh kg_Zn_^−1^. These values are also the best results comparing with many previous Zn‐air batteries using various non‐precious‐metal electrocatalysts (Table S2, Supporting Information).

**Figure 4:**
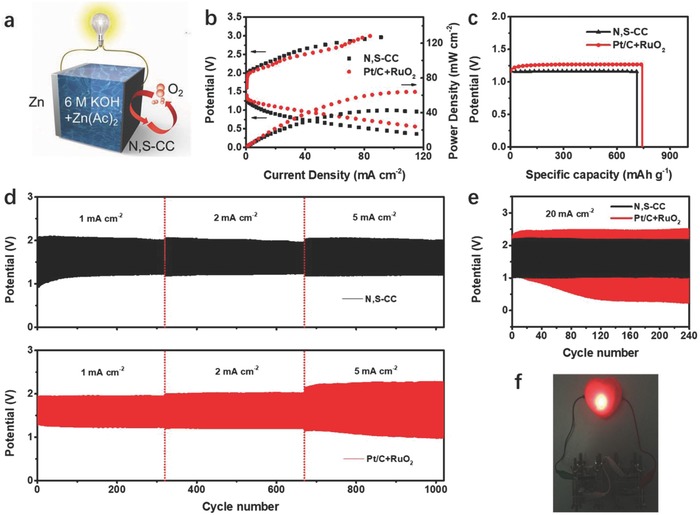
a) Schematic representation of liquid rechargeable Zn‐air batteries. b) Polarization and power density curves of batteries. c) Specific capacity of batteries at 5 mA cm^−2^. d) Cycling curves of rechargeable Zn‐air batteries with different catalysts, consecutively operated at 1 mA cm^−2^ for 320 cycles, 2 mA cm^−2^ for 350 cycles, and then 5 mA cm^−2^ for 350 cycles without interruption. The charge/discharge depth: 600 s cycle^−1^. e) Galvanostatic discharge–charge cycling curves at 20 mA cm^−2^. The charge/discharge depth: 600 s cycle^−1^. f) The photograph of a red‐heart‐shaped light powered by two Zn‐air batteries based on N, S‐CC catalyst in series.

The rechargeable performance of the two‐electrode battery using N, S‐CC was assessed by the charge/discharge cycling tests continuously operated at 1, 2, and then 5 mA cm^−2^ without interruption after initial few cycles' activation^7^ (Figure 4d). Evidently, when cycled at various current densities, our battery produces stable charge/discharge voltages with negligible voltage fading, signifying a superb recharge ability. The battery based on N, S‐CC exhibits a comparable charge/discharge potential in comparison to the counterpart using Pt/C+RuO_2_ at relative low current density (1, 2 mA cm^−2^). When cycled at higher current density (5 mA cm^−2^) after 350 cycles, our battery still achieves a charge potential of 2.0 V and a discharge potential of 1.2 V, with a high round‐trip energy efficiency of 60% and low voltage gap of only 0.8 V. In contrast, the battery based on Pt/C+ RuO_2_ presents much worse charge/discharge performance (a low energy efficiency of 43% and high voltage gap of 1.3 V). Notably, our two‐electrode battery with all‐in‐one N, S‐CC air electrodes runs stable over 1000 cycles in all (Figure 4d), much better than those of most recently reported two‐electrode Zn‐air batteries using various powdery catalysts such as N, P‐codoped carbon foam (180 cycles),^4^ P, S‐codoped C_3_N_4_ (200 cycles),^27^ defective graphene (95 cycles),^6^ N‐doped graphene nanoribbons (150 cycles),^26^ CuCo_2_O_4_@C (160 cycles),^25^ CoO/N‐doped carbon nanotubes (180 cycles),^10^ and Co/N/O tri‐doped graphene mesh (≈180 cycles)^7^ at similar current densities (0.5–5 mA cm^−2^). Notably, even cycled at much higher current density of 20 mA cm^−2^, our battery still operates fairly stable over 240 cycles (Figure 4e), while the Pt/C+RuO_2_ based cell suffers from severe stability decay probably due to the catalysts' deactivation by the alternative reduction/oxidation potential in a two‐electrode setup.^4^ In addition, our battery is also much superior to many previous results based on various bifunctional catalysts such as Fe‐Co‐N‐carbon (20 cycles),^40^ P‐doped C_3_N_4_ (50 cycles),^17^ and N‐doped CNT/CoO‐NiO‐NiCo (100 cycles)^41^ under similar conditions. Taken together, the above results manifest the excellent bifunctional ORR/OER activities and extraordinary durability of integrated N, S‐CC electrodes under practical operation conditions. Two Zn‐air batteries with N, S‐CC can be integrated into circuits to successfully power a red heart‐shaped light to work continuously over 12 h without brightness fading (Figure 4f) and light up various light‐emitting diodes (LEDs) with operation voltages of 2–3 V (Figure S23, Supporting Information).

We further constructed a flexible all‐solid‐state Zn‐air battery wherein N, S‐CC as the additive‐free air electrode was placed next to a piece of zinc foil coated by alkaline gel electrolyte (**Figure** **5**a). The assembled solid‐state battery indicates an excellent flexibility and a high open‐circuit voltage up to 1.25 V (Figure 5b). As depicted in Figure 5c, the as‐prepared N, S‐CC delivers comparable charge/discharge performance with that of Pt/C+RuO_2_ and a high peak power density of 47 mW cm^−3^. When discharged at constant current density of 5 mA cm^−3^, both of them show similar potential plateau (Figure 5d). These results further certify that N, S‐CC could also exhibit high catalytic performance close to the precious metal catalyst in all‐solid‐state batteries as well. Achieving a stable electrochemical performance under various mechanical deformations is vital for flexible electronic devices. Thus, we further perform the mechanical flexibility and cycling stability test of N, S‐CC based battery at 5 mA cm^−3^. Clearly, when the solid‐state device is bent to 90° and 135°, the discharge potentials show rather minor variations compared to that of the flat battery (0°), signifying a robust mechanical integrity (Figure 5e). Moreover, the charge/discharge cycles for single solid‐state battery can be maintained 120 cycles at 5 mA cm^−3^ under different bending conditions (Figure 5f). The demonstrated performance metrics are superior or comparable to many previous results from flexible Zn‐air batteries (Table S3, Supporting Information). As a demo, two solid‐state batteries connected in series can be utilized to drive a blue LED watch (Figure 5g; Movie S2, Supporting Information). The above results indicate attractive potential of N, S‐CC‐based solid‐state batteries in smart flexible electronics. The good ORR/OER performance of N, S‐CC is attributable to the following aspects: (1) each carbon fiber in N, S‐CC has unique shell–core structure wherein the internal intact core guarantees high conductivity, rendering sufficient electron transfer for active species in the shell; (2) the large surface area with hierarchical meso‐microporosity facilitates access of active sites and enhanced mass transport and unique porous structure renders N, S‐CC with enhanced available active surface area (10.93 cm^2^) relative to pristine CC (1.928 cm^2^), as derived from the electrochemical double‐layer capacitance results (Figure S24, Supporting Information); (3) N, S‐CC contains 3.93 at% N and 3.32 at% S, inducing rich reversible oxygen catalytic sites while N, S dual‐doping leads to asymmetrical spin and charge density, engendering a synergistic effect to improve the ORR/OER activity;^20^ (4) all‐in‐one electrode is free from pulverization and exfoliation issues associated with traditional powdery catalysts, beneficial for enhanced activity and stability.

**Figure 5:**
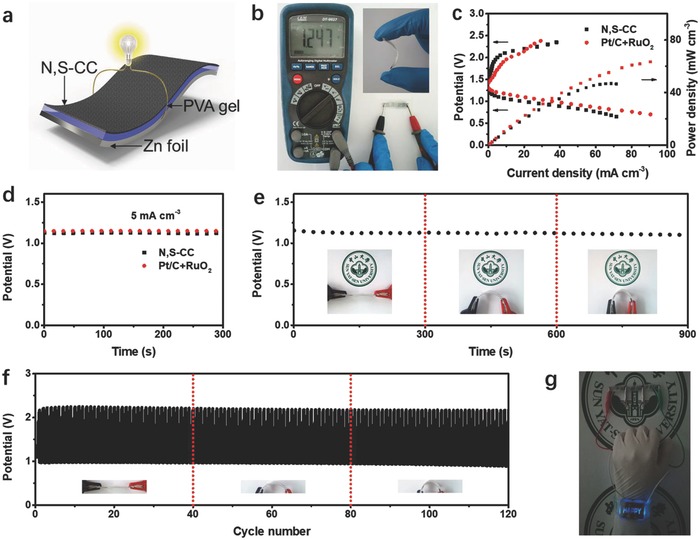
a) Schematic representation of an assembled solid‐state rechargeable Zn‐air battery with an N, S‐CC air electrode. b) Photograph of a solid‐state battery (packaged with a piece of parafilm) with an open‐circuit potential of 1.25 V. The inset shows the flexibility of the battery. c) Polarization and power density curves of batteries with N, S‐CC and Pt/C+RuO_2_ as catalysts. d) Galvanostatic discharge curves at 5 mA cm^−3^. e) Galvanostatic discharge curves at 5 mA cm^−3^ of N, S‐CC based battery under different bending conditions. f) Galvanostatic discharge–charge cycling curves at 5 mA cm^−3^ under different bending conditions. The charge/discharge depth: 240 s cycle^−1^. g) Photograph of a blue LED watch powered by two N, S‐CC based batteries in series.

In conclusion, we have developed a judicious in situ strategy to transform commercial carbon cloth into low‐cost, efficient, self‐standing, and ORR/OER bifunctional catalysts for the direct utilization as robust and additive‐free air electrodes in rechargeable Zn‐air batteries. Our strategy not only structures the smooth surface of carbon cloth into hierarchical meso‐microporous network for drastically increased catalytic surface area, but also realizes efficient S, N codoping for enriched ORR/OER active sites, while retaining intrinsically superb charge‐transfer ability and stable monolithic structures. Thus, our activated carbon cloth without loading any other active materials produces good ORR/OER activities and durability in one single material with a much smaller reversible overpotential (0.87 V) than those of Pt/C (1.10 V) and RuO_2_ (1.02 V) as well as many existing metal‐free bifunctional catalysts. When using activated materials directly as additive‐free air electrodes, as‐assembled liquid two‐electrode rechargeable Zn‐air batteries achieve a high energy efficiency of 60%, a specific capacity of 715 mAh g_Zn_^−1^, and a high density of ≈829 Wh kg_Zn_^−1^, along with long cycle life over 1000 cycles, surpassing most reported two‐electrode Zn‐air systems with those powdery carbon catalysts. Importantly, as‐fabricated flexible solid‐state cells also exhibit good rechargeable performance and high mechanical stability, promising for portable/wearable electronic devices. Not limited to Zn‐air batteries, our activated carbon cloth can find other applications such as fuel cells and water electrolysis. Our findings greatly broaden the application scope of such raw carbon materials and open up exciting avenues for developing next generation of low‐cost, environmental‐friendly, and flexible energy devices. The proposed strategy and design concept may spur the development of many other advanced self‐supporting electrodes for various energy conversion reactions.

## Conflict of Interest

The authors declare no conflict of interest.

## Supporting information

Supplementary

Supplementary

Supplementary
