# Supplementary material for: In Situ Activating Strategy to Significantly Boost Oxygen Electrocatalysis of Commercial Carbon Cloth for Flexible and Rechargeable Zn‐Air Batteries
Source: Adv Sci (Weinh). 2018 Oct 18;5(12):1800760. doi: 10.1002/advs.201800760 (PMC6299824; doi:10.1002/advs.201800760)
Supplement: Supplementary file 1 — Supplementary [file ADVS-5-1800760-s002.pdf]

Copyright WILEY-VCH Verlag GmbH & Co. KGaA, 69469 Weinheim, Germany, 2018.

ADVANCED SCIENCE
Open Access

Supporting Information

for *Adv. Sci.,* DOI: 10.1002/advs.201800760

In Situ Activating Strategy to Significantly Boost Oxygen Electrocatalysis of Commercial Carbon Cloth for Flexible and Rechargeable Zn-Air Batteries

Zhe Zhao, Zhongke Yuan, Zhengsong Fang, Junhua Jian, Jing Li, Meijia Yang, Chunshao Mo, You Zhang, Xuanhe Hu, Ping Li, Shuangyin Wang, Wei Hong, Zhikun Zheng, Gangfeng Ouyang, Xudong Chen, and Dingshan Yu**

Supporting Information

In Situ Activating Strategy to Significantly Boost Oxygen Electrocatalysis of Commercial Carbon Cloth for Flexible and Rechargeable Zn-Air Batteries

Zhe Zhao, Zhongke Yuan, Zhengsong Fang, Junhua Jian, Jing Li, Meijia Yang, Chunshao Mo, You Zhang, Xuanhe Hu, Ping Li, Shuangyin Wang, Wei Hong, Zhikun Zheng, Gangfeng Ouyang, Xudong Chen and Dingshan Yu**

Z. Zhao, Z. Yuan, Z. Fang, J. Jian, J. Li, M. Yang, C. Mo, Y. Zhang, X. Hu, P. Li, Prof. W. Hong, Prof. Z. Zheng, Prof. X. Chen* and Prof. D. Yu*

Key Laboratory for Polymeric Composite and Functional Materials of Ministry of Education and Key Laboratory of High Performance Polymer-based Composites of Guangdong Province

School of Chemistry

Sun Yat-Sen University

Guangzhou 510275, China

* E-mail: cescxd@mail.sysu.edu.cn, yudings@mail.sysu.edu.cn

Prof. G. Ouyang,

School of Chemistry

Sun Yat-Sen University

Guangzhou 510275, China

Prof. S. Wang

College of Chemistry and Chemical Engineering

Hunan University, Changsha, 410082, China

[+] These authors contributed equally to this work.

Synthesis of N, S-codoped Porous Carbon Cloth (N, S-CC)

Typically, first of all, a piece of clean carbon cloth (CC) was soaked in 15 mL of the mixture solution containing 1.25 mmol of zinc nitrate and 2.5 mmol of potassium hydroxide. The mixture solution with CC immersed inside was kept stirring for 8 hours and was subsequently transferred into a Teflon-lined stainless steel autoclave for the hydrothermal reaction at 160 °C for 18 hours. After completing the hydrothermal treatment, treated CC was washed by deionized water and dried under vacuum at 60 °C followed by the annealing treatment under Ar at 950 °C, which yielded *in situ* textured porous CC (T-CC). In the second step, 2 g of thiourea (THU) was dissovled in 20 mL of deionized water and T-CC was then immersed into the THU aqueous solution. The THU solution with T-CC was transferred into a Teflon-lined stainless steel autoclave for the hydrothermal reaction at 180 °C for 8 hours. After completing the hydrothermal treatment, treated T-CC was rinsed by deionized water and dried under vacuum at at 60 °C. Afterwards, treated T-CC was immersed into the ethanol solution containing benzyl disulfide (BDS, 50 mg/mL) with stirring for 1 hours, followed by rinsing and drying under vacuum at 60 °C. Finally, T-CC was loaded into a quartz tube and pyrolyzed under Ar at 900 °C for 1 hour with ramping rate 10 °C min^{-1}, producing S-doped porous T-CC (S-CC). In the third step, S-CC was was treated by NH_3 plasma (commercial RF source) with the optimal irradiation time of 25 min, the power of 90 W and the pressure of 20 Pa, eventually yielding N, S-codoped porous CC (N, S-CC).

Synthesis of S-codoped Porous Carbon Cloth (S-CC)

S-doped porous CC was prepared by the similar procedure mentioned above without NH_3 plasma. Typically, first, a piece of clean carbon cloth (CC) was soaked in 15 mL of the mixture solution of zinc nitrate and potassium hydroxide. The mixture solution with CC immersed inside was kept stirring for 8 hours and was subsequently transferred into a Teflon-lined stainless steel autoclave for hydrothermal reactions at 160 °C for 18 h. After hydrothermal treatment, treated CC was washed by deionized water and dried under vacuum at 60 °C followed by the annealing treatment under Ar at 950 °C, which yielded *in situ* textured porous CC (T-CC). In the second step, 2 g of thiourea (THU) was dissovled in 20 mL of deionized water and T-CC was then immersed into the THU aqueous solution. The THU solution with T-CC was transferred into a Teflon-lined stainless steel autoclave for the hydrothermal reaction at 180 °C for 8 h. After completing the hydrothermal treatment, treated T-CC was rinsed by deionized water and dried under vacuum at at 60 °C. Afterwards, treated T-CC was immersed into the ethanol solution containing benzyl disulfide (BDS, 50 mg/mL) with stirring for 1 hours, followed by rinsing and drying under vacuum at at 60 °C. Finally, T-CC was loaded into a quartz tube and pyrolyzed under Ar at 900 °C for 1 hour with ramping rate 10 °C min^{-1}, producing S-doped porous T-CC (S-CC).

Synthesis of N-doped Porous Carbon Cloth (N-CC)

S-doped porous CC was prepared by the similar procedure mentioned above without the step for S doping. Typically, first of all, a piece of clean carbon cloth (CC) was soaked in 15 mL of the mixture solution of zinc nitrate and potassium hydroxide. The mixture solution with CC immersed inside was kept stirring for 8 hours and was subsequently transferred into a Teflon-lined stainless steel autoclave

for hydrothermal reactions at 160 °C for 18 h. After hydrothermal treatment, treated CC was washed by deionized water and dried under vacuum at 60 °C followed by the annealing treatment under Ar at 950 °C, which yielded *in situ* textured porous CC (T-CC). In the second step, T-CC was was treated by NH_3 plasma (commercial RF source) with the optimal irradiation time of 25 min using the power of 200 W and pressure of 20 Pa, eventually yielding N-doped porous CC (N-CC).

Characterization

The morphology and microstructures for the samples were examined by scanning electron microscopy (SEM; Hitachi S-4800, Japan) equipped with an energy dispersive spectrometer (EDX) and transmission electron microscopy (TEM; JEM-2100F, Japan) operated at 200 kV. The X-ray photoelectron spectroscopy (XPS) experiments were carried out on Thermo ESCA Lab250 X-ray photoelectron spectrometer. The binding energies (BE) were calibrated by setting the measured BE of C 1s to 284.5 eV. The X-ray powder diffraction patterns were recorded on an X-ray diffractometer (XRD, Rigaku SmartLab, Japan). The nitrogen adsorption-desorption isotherms were measured at 77 K with ASAP 2460 system at 77 K. The specific surface areas were calculated by Brunauer−Emmett−Teller (BET) method, and the pore size distributions were derived by NLDFT method from the desorption branch of the isotherms. Raman spectra were recorded by Renishaw Raman system model 1000 spectrometer using a 20 mW air-cooled argon ion laser (514.5 nm) as the excitation source. The electron conductivity of all samples was measured by a standard four-probe method using RTS-9 system (Four Probes Tech, China). Water contact angles (θ) were performed by using a Kruss DSA 100 analysis system in a drop shape at ambient temperature. Each 3 µl water was automatically placed on the sample. This measurement repeated six times to obtain an average value.

Electrochemical Characterization

All electrochemical measurements were conducted with a CHI760E electrochemical workstation (Shanghai, China) in typical three-electrode cell. 1 M KOH aqueous solution saturated with oxygen/nitrogen was employed as the electrolyte for ORR and OER respectively.

The free-standing N, S-CC catalyst can be used directly as the working electrode for ORR and OER tests. For half-cell rotating disk electrode (RDE) and rotating ring-disk electrode (RRDE) tests, the N, S-CC electrode was produced by directly adhering the self-standing N, S-CC (cut to square piece of 3*3 mm) to a glassy-carbon electrode (GCE) with a diameter of 5 mm using Nafion (1 µL, wt 5 %) glue. As a control, commercial 20 wt% Pt/C or RuO_2 coated carbon cloth was also produced as follows: the catalyst ink containing Pt/C or RuO_2 was prepared by mixing 5 mg Pt/C or RuO_2, 50 µL of 5 wt% Nafion solution and 950 µL of ethanol under sonication for 1 hour. The catalyst ink was transferred onto the carbon cloth *via* a controlled drop casting method. The mass of the Pt/C or RuO_2 was controlled to achieve a loading amount of 0.3 mg cm^{-2}. A Pt foil electrode and a Ag/AgCl (saturated KCl solution) electrode were used as the counter electrode and reference electrode, respectively. Potentials measured against the reference electrode were calibrated manually against the reversible hydrogen electrode (RHE) according to the following equation:

$$E_{\mathrm{RHE}} = E_{\mathrm{Ag/AgCl}} + 0.204\ \mathrm{V} + 0.0592\ \mathrm{V} \times \mathrm{pH} \qquad (1)$$

Where E_{RHE} is the potential converted to the RHE and $E_{\mathrm{Ag/AgCl}}$ is the measured potential against the Ag/AgCl (saturated KCl solution) reference electrode, unless otherwise stated. Cyclic voltammograms (CVs) were performed at a scan rate of 50 mV s^{-1}, while linear sweep voltammetry (LSV) were recorded at a scan rate of 5 mV s^{-1} with 100 % IR-compensation (for OER testing) unless specifically mentioned. The RRDE testing was conducted on a RRDE with a GC disk (0.247 cm^2) and a Pt ring (0.186 cm^2) at 5 mV s^{-1} and a rotation rate of 1600 rpm. Based on RRDE results, the electron transferred number (n) and the percentage of HO^{2-} in total oxygen reduction products for ORR were calculated by using the followed equations:

$$n = 4\,\frac{I_d}{I_d + {}^{I_r}/_{N}} \qquad (2)$$

$$HO_2^- = 200\,\frac{{}^{I_r}/_{N}}{I_d + {}^{I_r}/_{N}} \qquad (3)$$

Where I_d is disk current, I_r is ring current, and N is current collection efficiency of the Pt ring, which was determined to be 0.40 from the reduction of $K_3Fe[CN]_6$.

Chronopotentiometry (E-t) testing in constant current (j = 10 mA cm^{-2}) were performed to evaluate the long-term stability. The impedance spectra (EIS) for OER was measured with the three-electrode system under 1.4 V vs. RHE over the frequency range from 1 MHz to 0.1 Hz in 1 M KOH.

The electrochemically active surface area (ECSA) was determined by measuring the capacitive current from double-layer charging according to the scan-rate dependence of cyclic voltammograms (CVs).[S1] Briefly, a potential range of -0.1 V – 0.1 V vs. Ag/AgCl with no obvious Faradaic processes was applied for CV in 1 M KOH solution and the charging current signal (i_c) was recorded at various scan rates (v). The (C_{DL}) was directly derived from the slope of i_c – ν plots, according to equation 4.

$$i_c = \nu C_{DL} \qquad (4)$$

Thus, the C_{DL} of NS-CC and CC were calculated to be 0.4372 and 0.07713 mF, respectively. Since the specific capacitance (Cs) value of carbon in 1 M NaOH was determined to be 0.040 mF cm-2 according to previous reports,[S1] the ECSA was determined with equation 5.

$$\mathrm{ECSA} = C_{DL} / C_s \qquad (5)$$

The ECSA of NS-CC and CC were calculated to be 10.93 and 1.928 cm^2 respectively.

Faraday efficiency was determined by using the volumetric method.[S2] Typically, the generated O_2 over N, S-CC was accumulated in a 10 mL graduated tube, filled with the KOH electrolyte. Current dominated electrolysis was executed at the current density of 10 mA cm^{-2} for 2 h under ambient conditions. At each 0.3 mL of O_2, the time was recorded. The collected charges going through working electrodes, in the meantime, were reckoned by current x time. The collected gas product was sampled with a Hamilton syringe and detected by using gas chromatograph (GC, Agilent 7820A) to evaluate its purity. According to the GC spectra, it was found that O_2 could be the only gaseous product detected, indicating the gas products had no other impurities.

Liquid Zn-air battery assembly: Liquid Zn-air batteries were tested in home built electrochemical cells. The carbon cloth-based catalysts (N, S-CC and carbon cloth loading Pt/C or Pt/C+RuO_2, the mass loading of precious catalyst is 0.3 mg cm^{-2}) were cut into 1.2 ×1.2 cm^2, and then were attached to a gas diffusion layer (GDL) to form a hybrid electrode by hot-press method. This hydrophobic hybrid air electrode could effectively avoid the problem of liquid electrolyte flooding or leaking.[S2] The polished Zn foil (0.05 mm in thickness, Alfa Aesar) was used as the anode and the used electrolyte was 6 M KOH containing 0.2 M zinc acetate. The time of each charge/discharge cycle is 600 s. All the measurements were carried out using a CHI760E electrochemical workstation and a LAND CT2001A multi-channel battery testing system. The specific capacity and energy density are derived from the galvanostatical discharge curves, normalized to the mass of consumed Zn electrodes.

All-solid-state Zn–air battery assembly: A piece of activated carbon cloth (N, S-CC, working area: 0.5 cm^2) can be directly used as the air electrode without any binders and extra current collectors. A piece of polished zinc foil (0.05 mm in thickness) was ultilized as the anode. The gel polymer electrolyte was produced as below: 1 g of PVA powder (MW 195000, Aladdin) and 0.1 g of PEO powder (MW 100000, Alfa Aesar) was dissolved in 10 mL of deionized water at 95 °C under magnetic stirring for about 2 h. Afterwards, 1 mL of 18 M KOH solution was added and the mixture solution was kept stirring at 95 °C for about 40 min. Then the solution was freezed at -20 °C for 3 h, and then thawed at room temperature. Finally, the activated carbon cloth N, S-CC and zinc foil were placed tightly on the two sides of gel polymer electrolytes without any additives. The time of each charge/discharge cycle is 240 s. All the measurements were carried out on the as-assembled solid-state cell under atmoshpere condition by using a CHI760E electrochemical workstation and a LAND CT2001A multi-channel battery testing system.

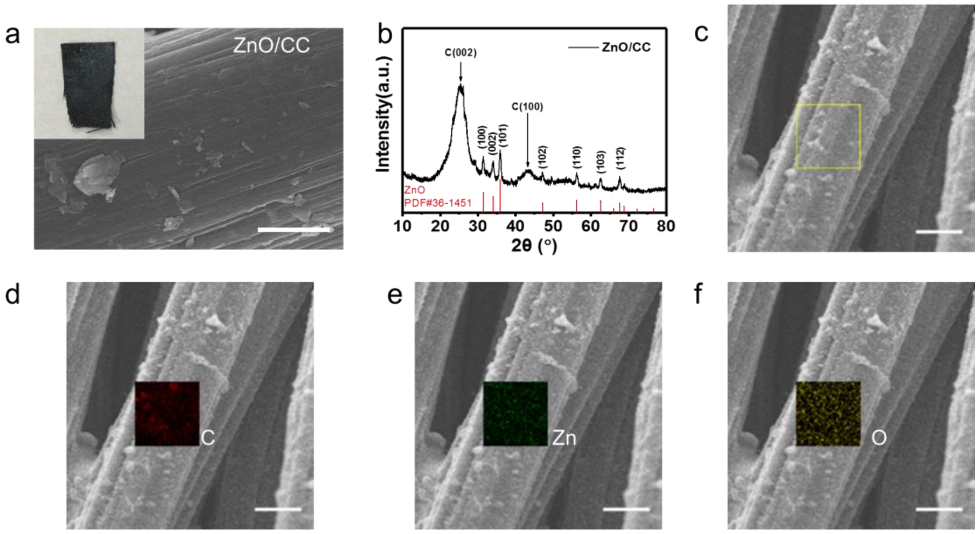

Figure S1. (a) SEM image of ZnO coated carbon cloth (ZnO/CC, scale bar: 2 μm). The inset shows the optical photograph. (b) Powder XRD patterns of ZnO/CC. (c-f) Selected SEM image of ZnO coated carbon cloth (c) with EDX element mapping of C (inset of d) , Zn (inset of e), and O (inset of f).

The rough surface on the fiber wall from SEM observation and the obvious XRD peaks from ZnO in XRD patterns verify the successful coating of ZnO on the carbon cloth. The images of EDX element mapping further indicate the homogenous dispersion of ZnO on the carbon cloth.

The ZnO-assisted structuring is the first step of our activating process to create porous surface structure. The loading amount of ZnO indeed affects the textural structure of activated carbon cloth, which is generally related to the electrode performance. Indeed, we also did some controlled experiments. It was found that relatively low loading of ZnO leads to a low specific surface area (< 50 m^2/g), while overhigh ZnO loading severely destructs the carbon fiber structure, which compromises the mechanical flexibility of activated carbon cloth, undesirable for self-supportive electrodes.

Considering the balance between the surface area and mechanical flexibility, we carefully chose the optimal conditions in our present work.

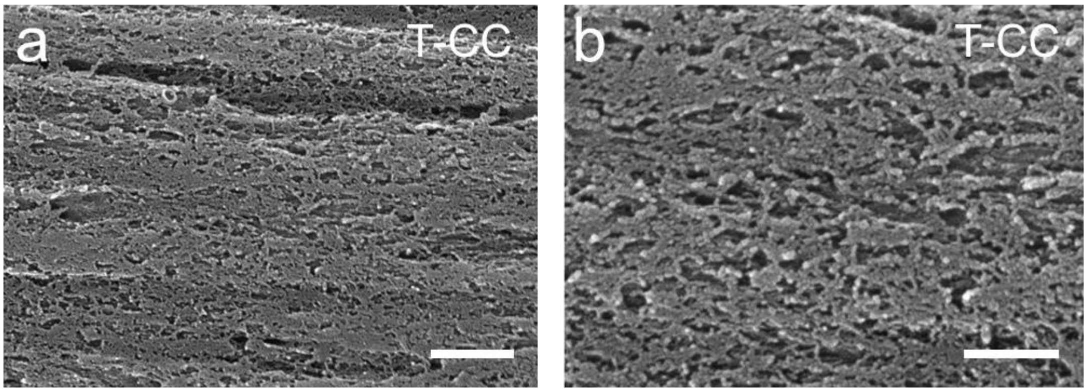

Figure S2. Typical SEM image (a) and enlarged SEM image (b) of T-CC. Scale bar: 500 nm (a) and 250 nm (b), respectively.

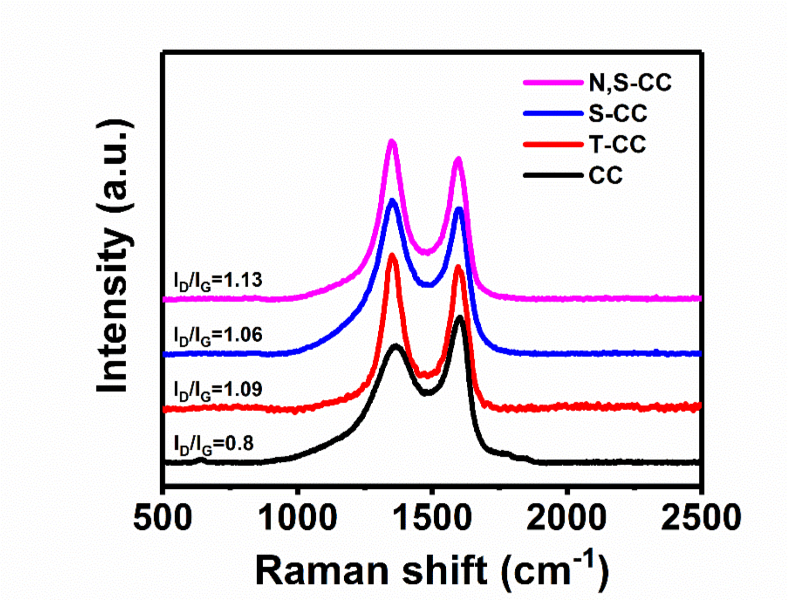

Figure S3. Raman spectra of CC, T-CC, S-CC and N, S-CC, respectively.

Figure S3 shows Raman spectra of CC after subjecting to each step of activation. Raman spectra of all the samples reveal the typical D and G peaks at 1350 and 1580 cm^{-1}, respectively. Generally,

the intensity ratio of D to G band (I_D/I_G) is indicative of the amount of defect sites and disordered structures. T-CC shows a higher I_D/I_G value relative to pristine CC, which is mainly caused by the formation highly porous structure on T-CC (Figure S2). After subsequent S doping into T-CC, the I_D/I_G value decreases, which lies in that the annealing treatment at 900 °C involved in this step could remove some oxygen-carrying groups and improve the graphitic degree of T-CC. When S-CC was subjected to NH_3 plasma treatment, the I_D/I_G value increases relative to S-CC, since NH_3 plasma not only introduces the extra N atoms into carbon framework, but also etches the CC surface and thereby introduce more defective sites.[S3]

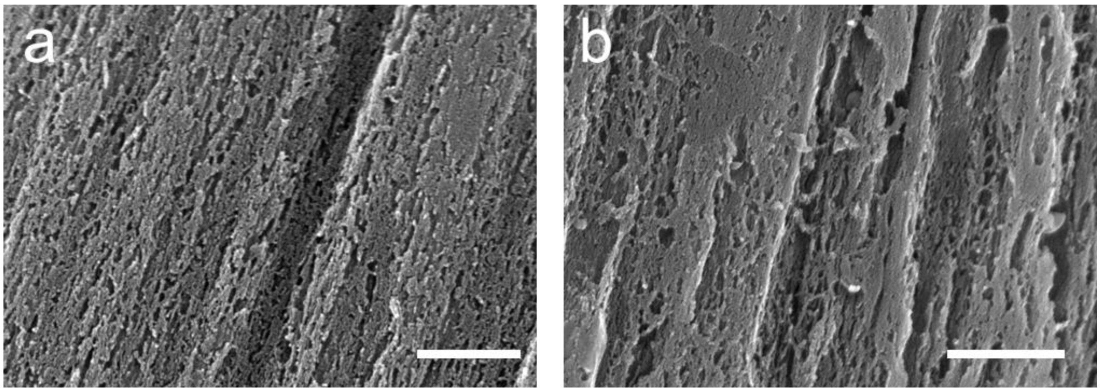

Figure S4. Typical SEM images of N-CC and S-CC, scale bar = 200 nm

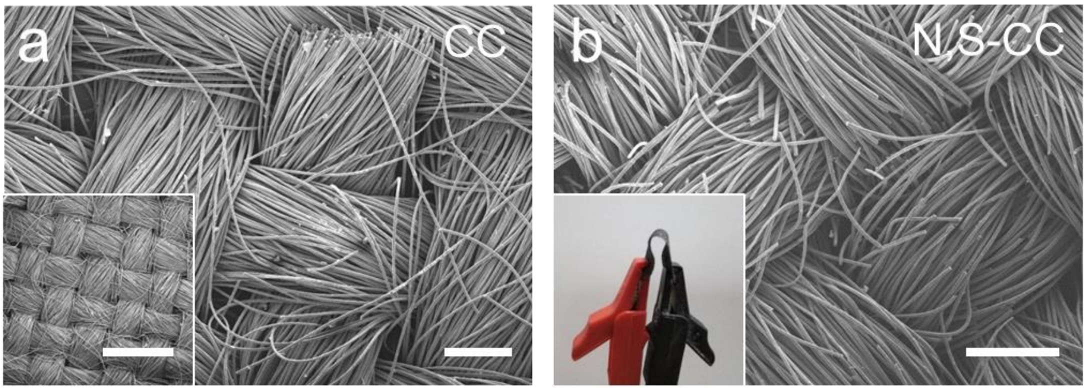

Figure S5. (a) SEM image of commercial carbon cloth, scale bar = 200 μm, inset scale bar = 1 mm. (b) SEM image of N, S-CC after reciprocating bending, scale bar = 200 μm. The morphology was maintained after in *situ* activation.

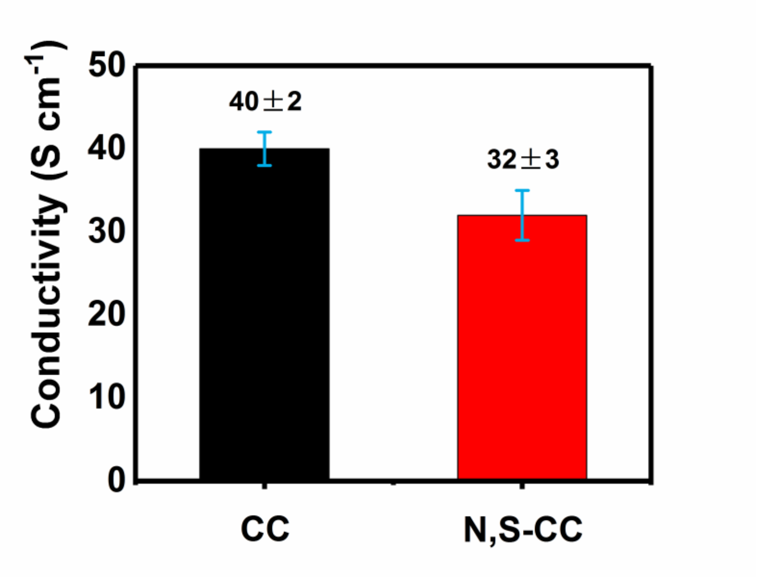

Figure S6. Electrical conductivity of CC and N, S-CC measured by using four-probe method. The average conductivity of CC and N, S-CC was derived from five specimens and error bars for the standard deviation are given.

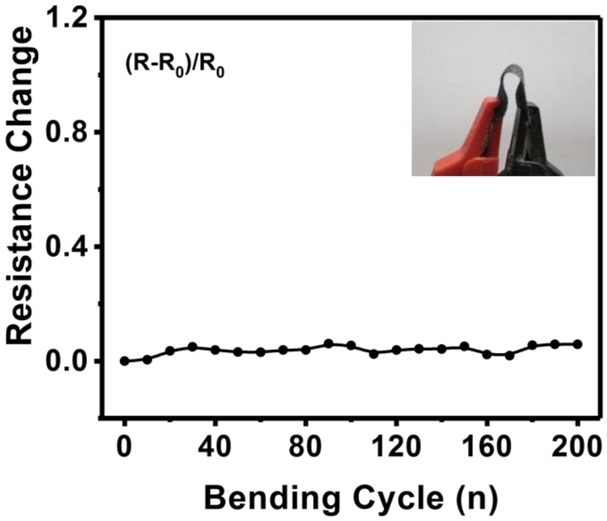

Figure S7. Electrical resistance change of N, S-CC when the sample was bent to 180º and then straightened for each cycle. The inset shows the bending process during measurements.

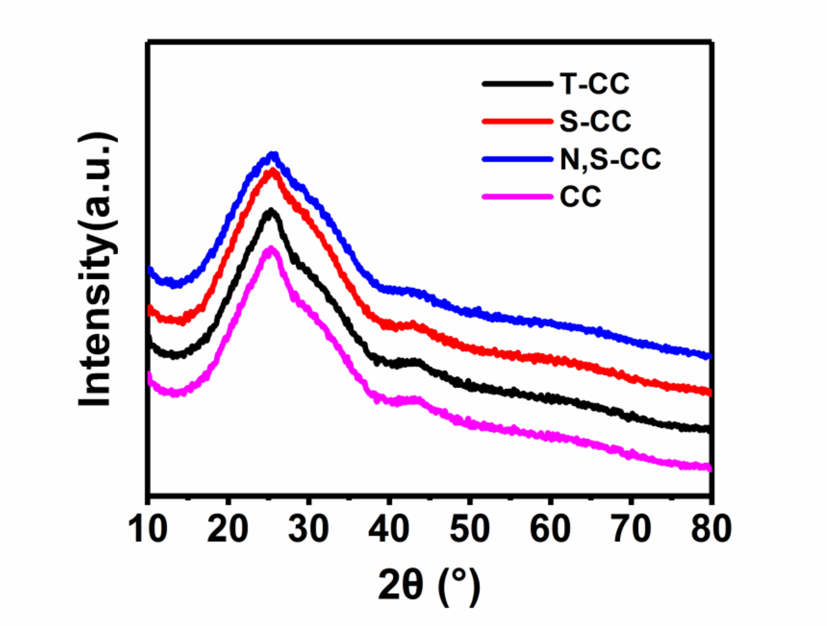

Figure S8. XRD patterns of CC, T-CC, S-CC and N, S-CC. All XRD patterns show two typical XRD peaks at 26° and 44°, corresponding to (002) and (100) peaks of carbon materials, respectively. As observed by X-ray diffraction analysis (Figure S8), upon each step of activation, the crystalline structure of CC gives a minor change relative to pristine CC.

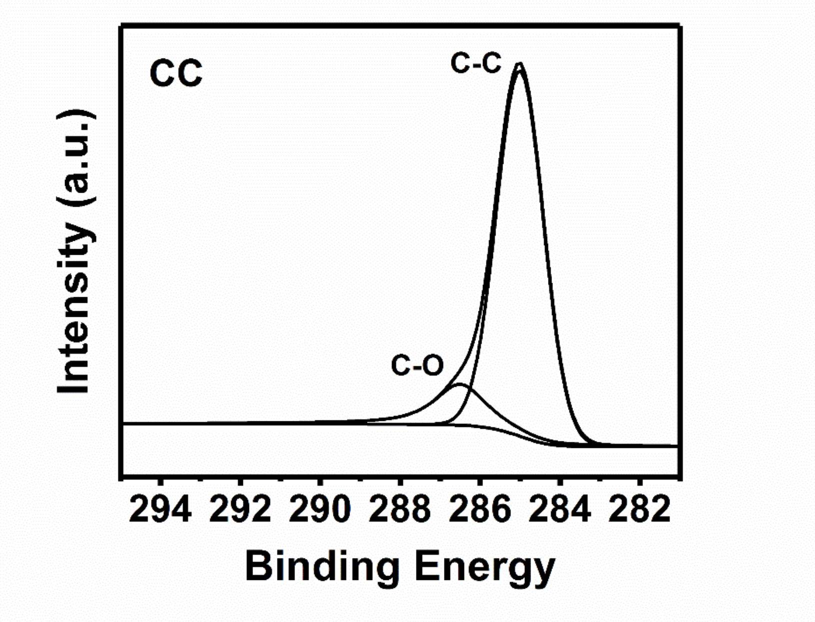

Figure S9. High resolution XPS C 1s spectra for CC.

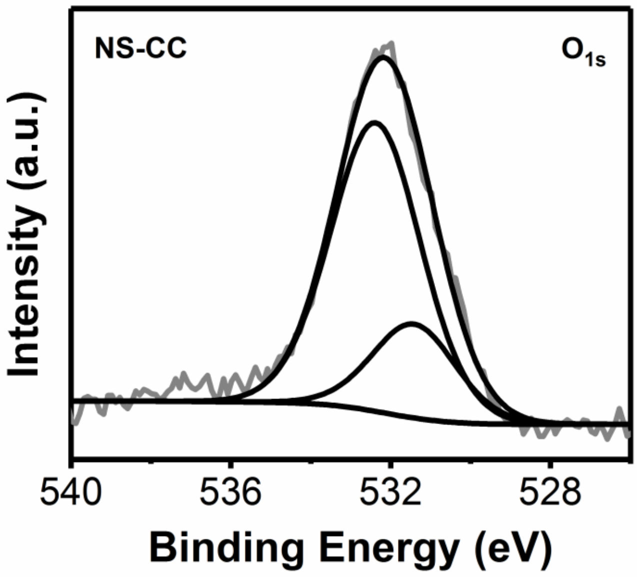

Figure S10. High-resolution XPS C 1s spectrum of N, S-CC. The O1s spectrum can be fitted into two components. The strong signal at 532.2 eV is attributed to the physically absorbed oxygen on the CNT surface due to air exposure,[S4] while the weak signal at 531.8 eV arises from the residual C-O groups on the N, S-CC.[S5] Also, XPS analysis indicates the oxygen content of N, S-CC is similar to that of pristine CC without any treatment. Taken together, the above results imply that after three-step activation, the oxygen-containing groups are largely removed.

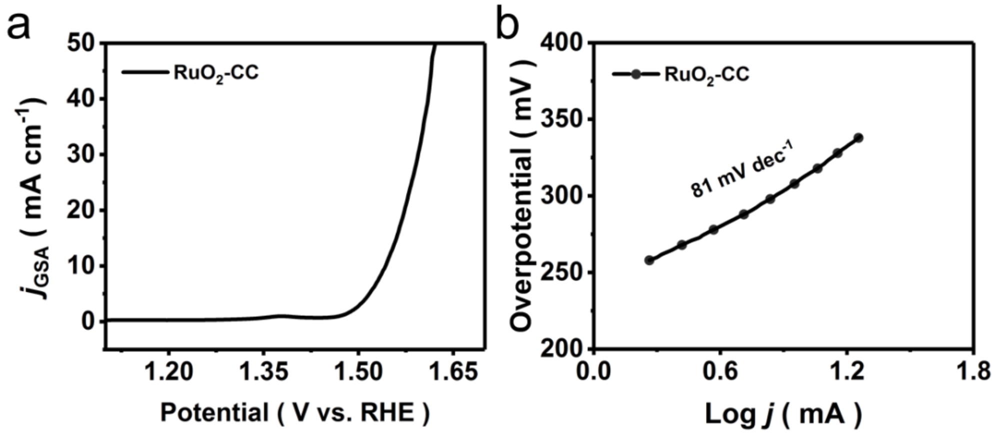

Figure S11. (a) LSV curve of commercial RuO_2 coated carbon cloth for OER in 1 M KOH. (b) The corresponding Tafel curve.

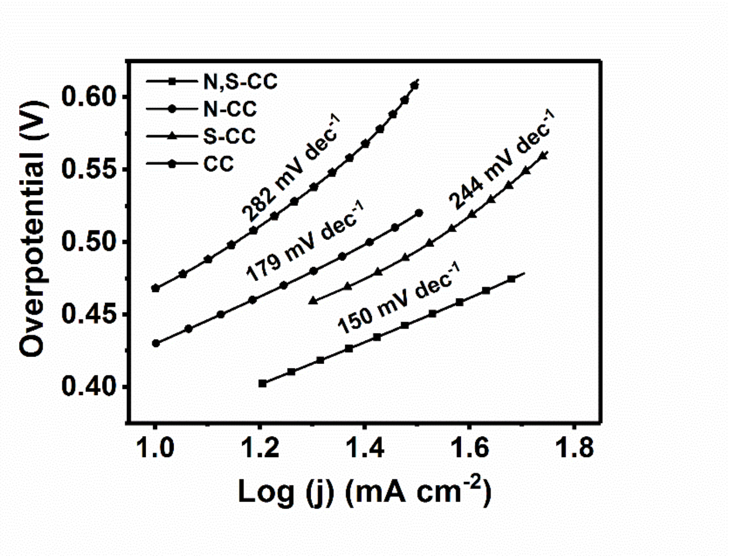

Figure S12. Tafel curves of CC, S-CC, N-CC and N, S-CC for OER in 1 M KOH.

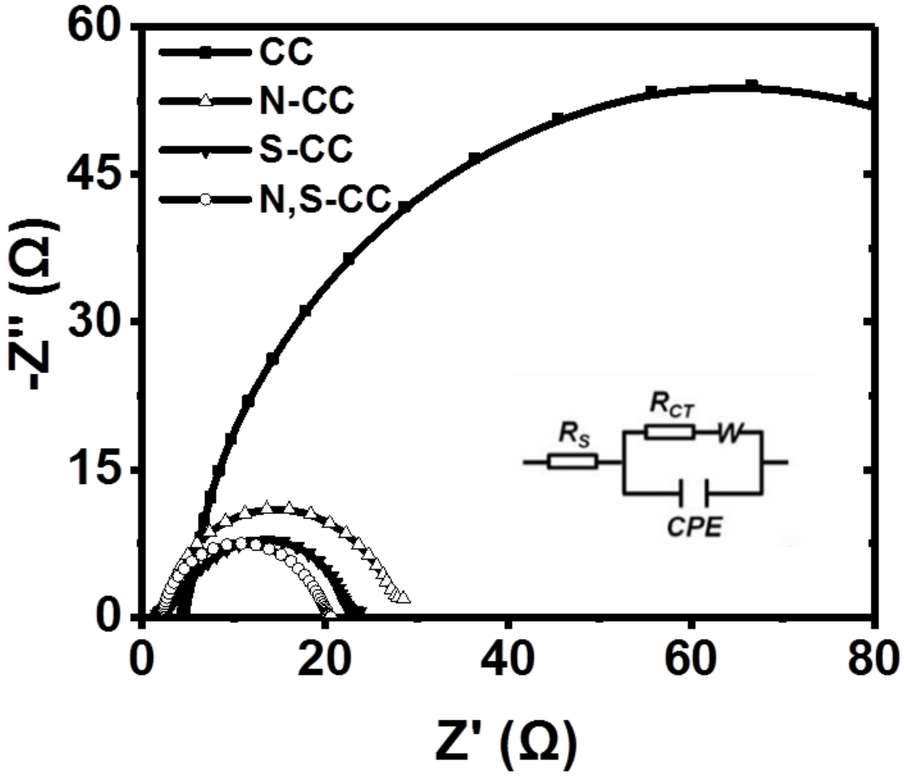

Figure S13. Nyquist plots for CC, S-CC, N-CC and N, S-CC at an overpotential of 270 mV.

The fitted data reveals the charge transfer resistances for the activated CC electrodes including

S-CC (R_{CT} = 21 Ω), N-CC (R_{CT} = 27 Ω) and N, S-CC (R_{CT} = 18 Ω), which are smaller than that of the CC electrode (R_{CT} = 125 Ω).

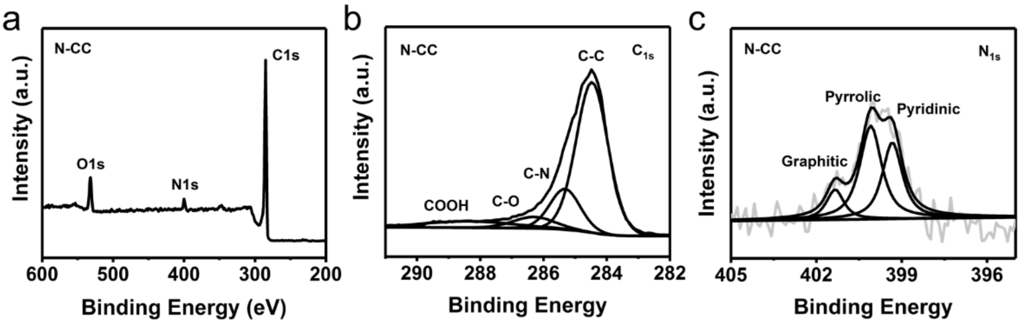

Figure S14. (a) XPS survey spectra of N-CC and the corresponding high-resolution XPS spectra of (b) C1s and (c) N 1s, respectively. XPS analysis reveals the N content of 3.61 at%, close to that of N, S-CC (4.23 at% N).

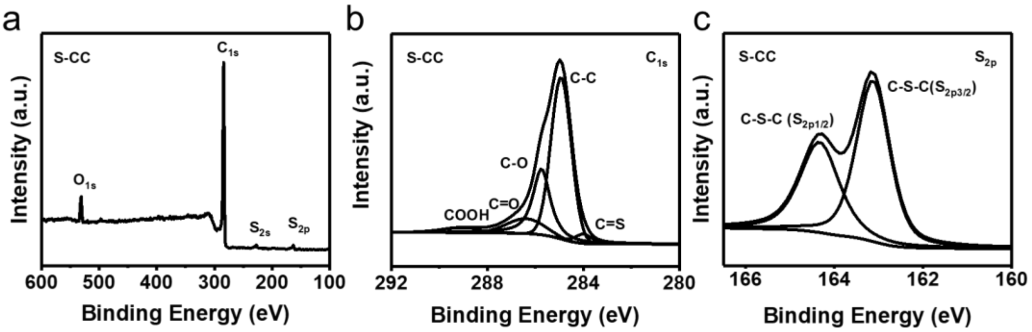

Figure S15. (a) XPS survey spectra of S-CC and the corresponding high-resolution XPS spectra of (b) C1s and (c) S 2p, respectively. XPS analysis reveals the S content of 2.77 at%, close to that of N, S-CC (3.02 at% S).

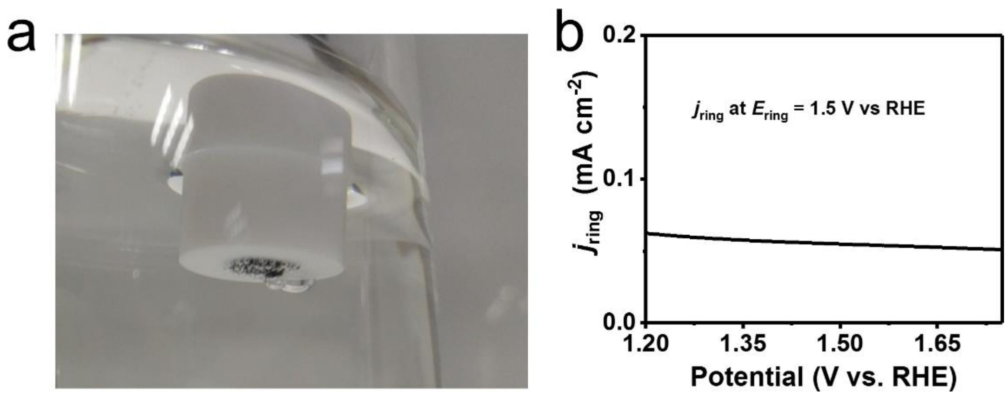

Figure S16. (a) Photograph of N, S-CC adhered onto rotating disk electrode using Nafion glue after LSV scan in the OER region, showing O_2-bubbles generated on the electrode surface. (b) Detection

of peroxide evolution from the N, S-CC electrode using RRDE measurements. The peroxide generated during the anodic polarization scan was reduced at the Pt ring at a constant potential of 1.5 V and the ring current signal was recorded.

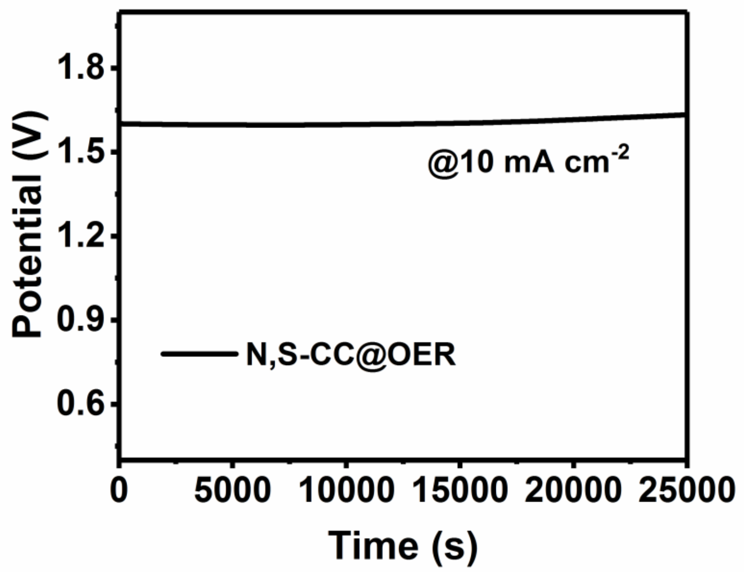

Figure S17. Chronopotentiometric curves obtained in constant current (j = 10 mA cm^{-2}) electrolysis using N, S-CC in 1 M KOH.

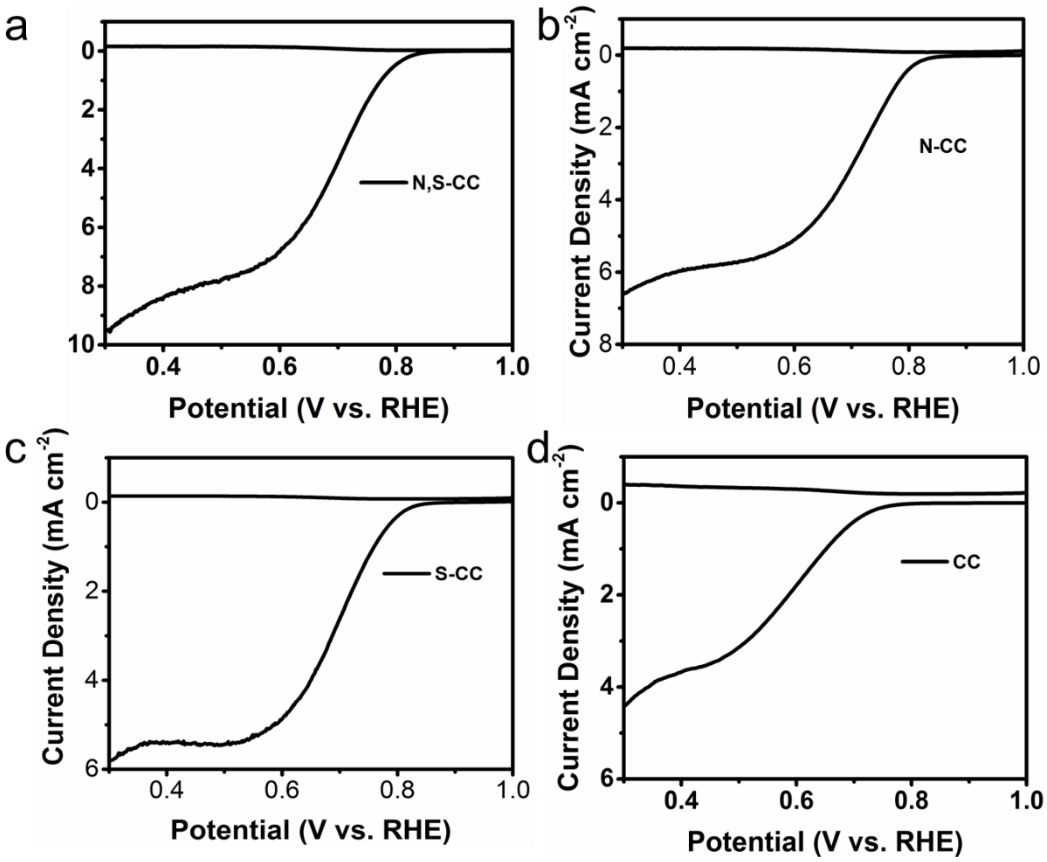

Figure S18. RRDE curves of (a) N,S-CC, (b) N-CC, (C) S-CC and (d) CC at the rotation rate of 1600 rpm in 1 M KOH.

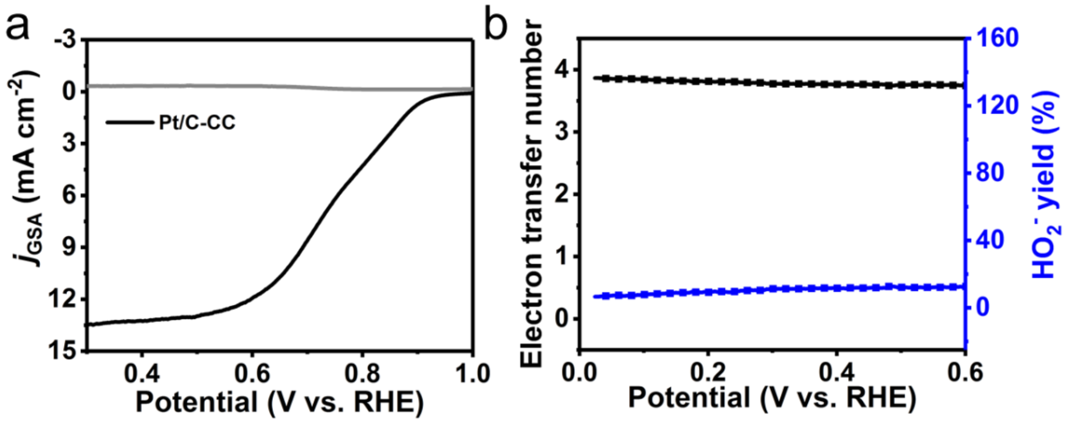

Figure S19. (a) RRDE curves of Pt/C coated carbon cloth for ORR at the rotation rate of 1600 rpm in 1 M KOH. (b) Electron transfer number (n) and the percentage of peroxide in the total oxygen reduction products derived from LSV curves in RRDE measurements in (a).

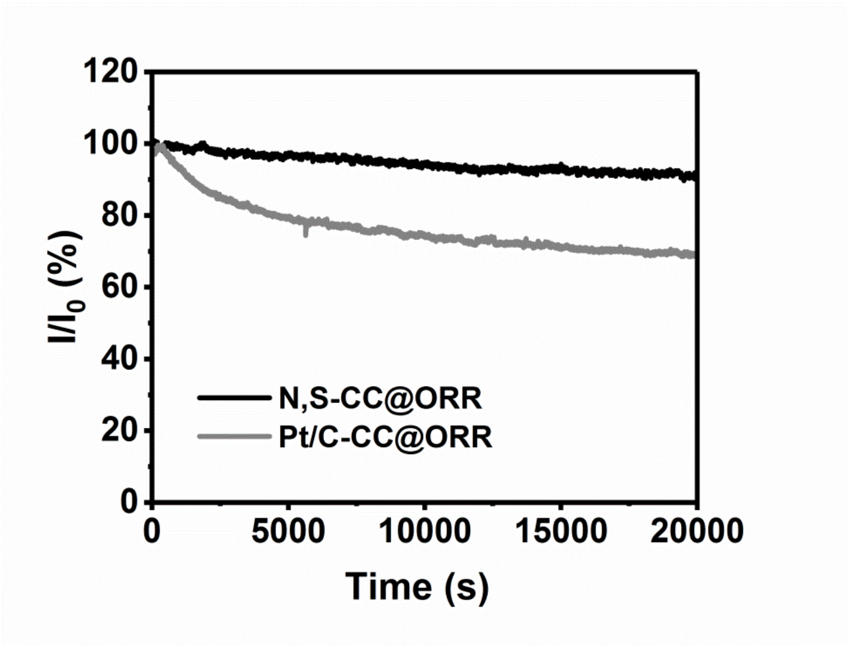

Figure S20. ORR chronoamperometric response of N, S-CC and commercial Pt/C coated CC at a constant potential of 0.6 V in 1 M KOH.

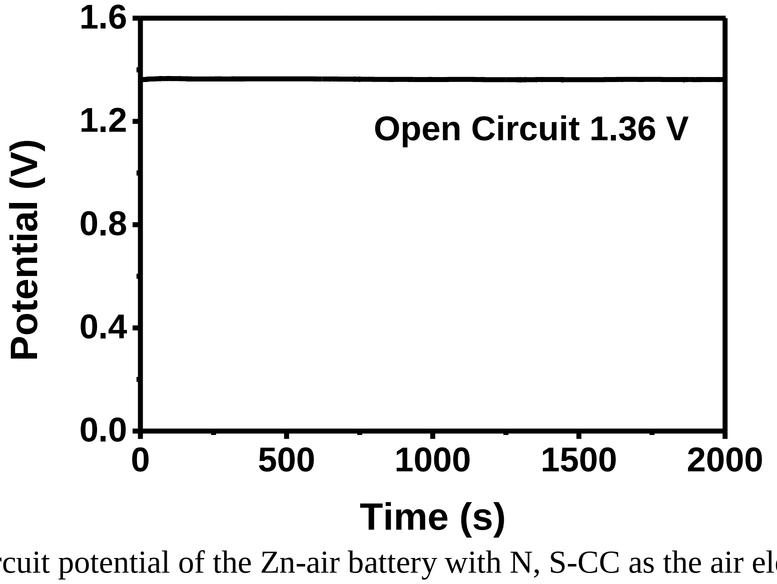

Figure S21. Open circuit potential of the Zn-air battery with N, S-CC as the air electrode.

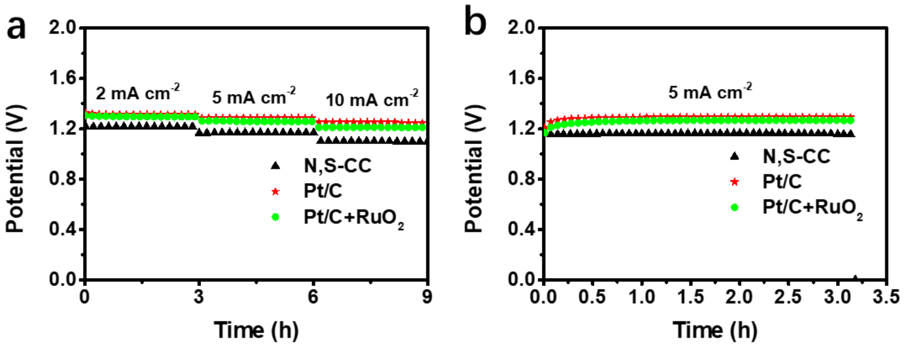

Figure S22. (a) Galvanostatic discharge curves of the Zn-air battery based on various catalysts at different current densities. (b) Galvanostatic discharge curves of Zn-air batteries at 5 mA cm^{-2}, corresponding to Figure 4c.

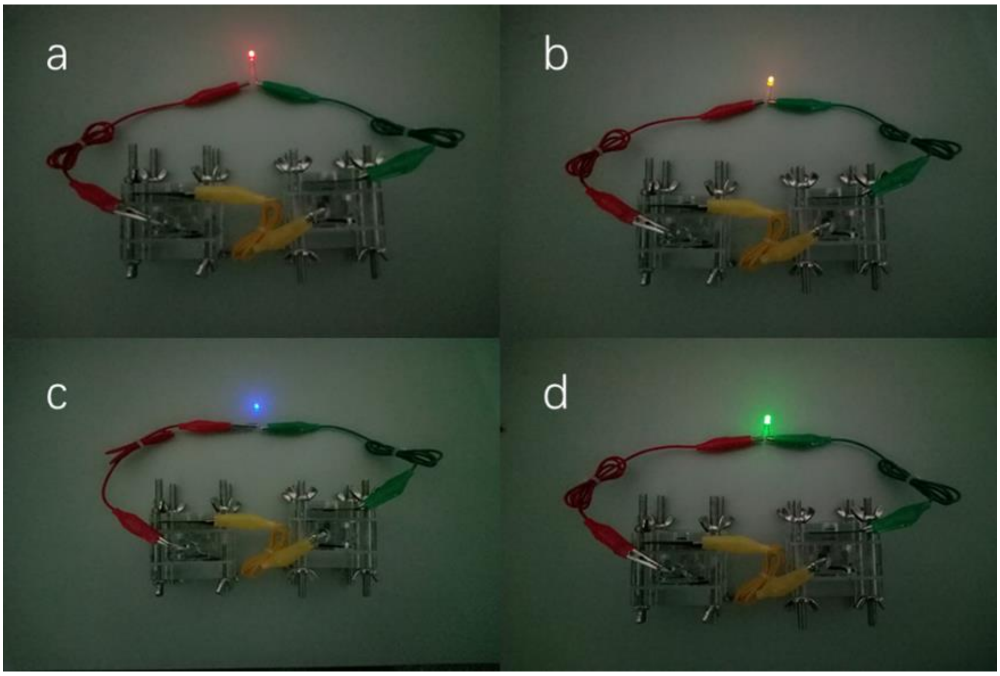

Figure S23. Photograph of various color of LED powered by two liquid Zn-air batteries with the N,S-CC cathode connected in series.

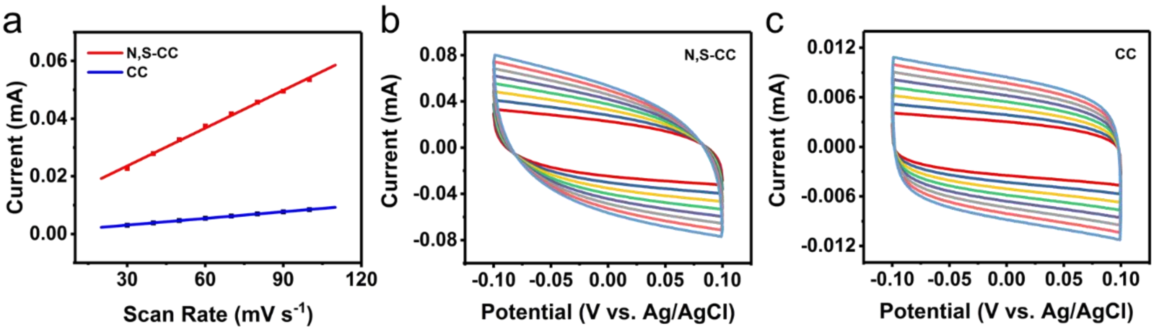

Figure S24. (a) The anodic capacitance currents plotted as a function of scan rate for N, S-CC and CC respectively. (b) Cyclic voltammograms obtains with N, S-CC in the capacitance current range (-0.1 V ~ 0.1 V vs. Ag/AgCl) at a scan rates of 30, 40, 50, 60, 70, 80, 90, 100, 110 mV s^{-1} in 1M KOH solution, respectively. (c) Cyclic voltammograms obtains with CC in the capacitance current range (-0.1 V ~ 0.1 V vs. Ag/AgCl) at various scan rates of 30, 40, 50, 60, 70, 80, 90, 100 mV s^{-1} in 1 M KOH solution, respectively.

Table S1. The OER performance of various self-supporting and powdery metal-free electrocatalysts in ***1 M KOH.***

Electrocatalysts	Mass loading (mg cm^{-2})	η@10mA cm^{-2} (mV)	Ref.
**N,S-CC**	**0.3**	**360**	**This work**
NPMC-1000	0.5	~520 @5mA	*Nat. Nanotechnol*. **2015**, 10, 444
N-Carbon nanotube framework	0.2	370	*Nat. Energy* **2016**, 1, 15006
ONPPGC/OCC	0.1	410	*Energy Environ. Sci.* **2016**, 9, 1210
N-doped GNS@Carbon paper	-	~380	*Chem. Commun.*, **2017**, 53, 7748
C_3N_4–CNT–CF	0.5	410	*J. Mater. Chem. A* **2016**, 4, 12878
NiD-PCC	8	360	*Energy Environ. Sci.***2016**, 11, 3411
DRPC	0.16	360	*J. Mater. Chem. A* **2017**, 5, 17064
N-GRW	0.6	360	*Sci. Adv.* **2016**, 2, e1501122
NSGF	-	346	*Adv. Energy Mater.* **2016**, 6, 1501492
S,S'-CNT	0.23	350	*Adv. Energy Mater.* **2016**, 6, 1501966

Note: All the OER performance was recorded in ***1 M KOH*** derived from the literatures.

Table S2. The performance of liquid ***two-electrode*** Zn-air batteries with various electrocatalysts.

Electrocatalysts	Mass loading (mg cm^{-2})	Peak power density (mW cm^{-2})	Specific capacity (mAh g^{-1})	Energy density (mWh g^{-1})	Charge/discharge voltage gap (V@mA cm^{-2})	Stability	Ref.
**N,S-CC**	**0.3**	**42**	**715**	**829**	**~0.80@5**	**@600 s/cycle for 1020 cycles**	**This work**
Defect graphene	0.1	15.4	-	-	-0.77@1	@300 s/cycle for 95 cycles	*Adv. Mater.* **2016**, 28, 9532
CoO/N-CNT	1.0	~265	~570	>700	~0.60@5	@ 200 s/cycle for 60 cycles	*Nat. Commun.* **2013**, 4, 1805
Ag-Cu on Ni foam	0.8	86	572	641	0.96@20	@1200 s/cycle for 100 cycles	*Electrochimica Acta*, **2015**, 158, 437
CoZn-NC-700	1.2	-	578	694	0.73~1.10 @10	@600 s/cycle for 385 cycles	*Adv. Funct. Mater.* **2017**, 27, 1700795
NCNT/$Co_xMn_{1-x}O$	0.53	81	581	695	0.57@7	@600 s/cycle for 72 cycles	*Nano Energy*, **2016**, 20, 315
NCNT/CoO-NiO-NiCo	0.53	102	~594	~713	0.86@20	@600 s/cycle for 100 cycles	*Angew. Chem. Int. Ed.* **2015**, 54, 9654
$CuCo_2O_4$@C	1	~60	-	-	~0.6-0.8@2	@1800s/cycle for 160 cycles	*Nano Lett.* **2017**, 17, 7989
Meso/micro-FeCo-Nx-30	2	150	-	-	0.80@10	@7200s/cycle for 20 cycles	*Angew. Chem. Int. Ed.* **2018**, 57, 1856
$Co(OH)_2$+N-rGO	1	36	-	-	1.29@15	@2400 s/cycle for 75 cycles	*ACS Appl. Mater. Interfaces*, **2015**, 7, 12930
P-doped C_3N_4/CFP	0.2	-	-	-	~1.40@20	@600 s/cycle for 50 cycles	*Angew. Chem. Int. Ed.* **2015**, 54, 4646
Co_3O_4 nanowires /stainless steel	1.5	40	-	-	1.16@50	@600s/cycle for 100 cycles	*Adv. Energy Mater.* **2014,** 4, 1301389
CNT/graphene	0.5	-	~712	~872	-	-	*Adv. Mater.* **2016**, 28, 4606
NPMC-1000	0.5	~55	~735	~835	1.75@2	@600 s/cycle for 180 cycles	*Nat. Nanotechnol.* **2015**, 10, 444
NGM-Co	0.5	152	750	840	~1.00@2	@1200s/cycle for 180 cycles	*Adv. Mater.* **2017,** 29, 1703185

Note: The listed performance metrics were measured in ***two-electrode*** Zn-air batteries.

Table S3. The performance of all-solid-state Zn-air batteries with various electrocatalysts.

Electrocatalysts	Mass loading (mg cm^{-2})	Peak power density (mW cm^{-3})	Substrate	stability	Ref.
**N,S-CC**	**0.3**	**47**	**None**	**@5 mA cm^{-3}, 240 s/cycle for 120 cycles**	**This work**
N-Co_3O_4	~5.8	32	Carbon cloth	@2.5 mA cm^{-3},4800 s/cycle for 21 cycles	*Adv. Mater.* **2017**, 29. 1602868
NGM-Co	1.5	-	Carbon cloth	@2 mA cm^{-2}, 200 s/cycle for 18 cycles	*Adv. Mater.* **2017**, 29. 1703185
Nanoporous CNF	None	-	None	@2 mA cm^{-2}, 635 s/cycle for 34 cycles	*Adv. Mater.* **2016**, 28, 3000
Ultrathin Co_3O_4/CC	0.3	<20(mW cm^{-2})	Carbon cloth	@2 mA cm^{-2}, 720s/cycle for 30 cycles	*Adv. Energy Mater.* **2017**, 1700779
NiO/CoN PINWs	-	-	None	@1 mA cm^{-2}, 600s/cycle for 50 cycles	*ACS Nano* **2017**, 11, 2275

Supplementary References:

[S1] a) X. Lu, W. Yim, B. Suryanto, C. Zhao, *J. Am. Chem. Soc.* **2015**, *137*, 2901; b) C. McCrory, S. Jung, J. Peters, T. Jaramillo, *J. Am. Chem. Soc.* **2013**, *135*, 16977.

[S2] H. B. Yang, J. W. Miao, S. F. Hung, J. Z. Chen, H. B. Tao, X. Z. Wang, L. P. Zhang, R. Chen, J. J. Gao, H. M. Chen, L. M. Dai, B. Liu, *Sci. Adv.* **2016**, 2: e1501122.

[S3] X. Li, H. Wang, J. T. Robinson, H. Sanchez, G. Diankov, H. Dai, *J. Am. Chem. Soc.* **2009**, *131*, 15939.

[S4] H. Li, T. Xu, C. Wang, J. Chen, H. Zhou, H. Liu, *Diam. Relat. Mater.* **2006**, *15*, 1228.

[S5] a) J. L. Hueso, J. P. Espinós, A. Caballero, J. Cotrino, A. R. González-Elipe, *Carbon* **2007**, *45*, 89. b) S. Biniak, G. Szymański, J. Siedlewski, A. Świątkowski, *Carbon* **1997**, *35*, 1799.
